# Obese living kidney donors: a comparison of hand-assisted retroperitoneoscopic versus laparoscopic living donor nephrectomy

**DOI:** 10.1007/s00464-019-07276-x

**Published:** 2019-11-18

**Authors:** Kosei Takagi, Hendrikus J. A. N. Kimenai, Jan N. M. IJzermans, Robert C. Minnee

**Affiliations:** 1 Division of HPB & Transplant Surgery, Department of Surgery, Erasmus MC, University Medical Centre Rotterdam, Dr. Molewaterplein 40, 3015 GD Rotterdam, The Netherlands; 2 Department of Gastroenterological Surgery, Okayama University Graduate School of Medicine, Dentistry, and Pharmaceutical Sciences, Okayama, Japan

**Keywords:** Kidney transplantation, Living donors, Nephrectomy, Laparoscopy, Hand-assisted laparoscopy, Obesity

## Abstract

**Background:**

The aim of this study was to examine the difference in outcome between hand-assisted retroperitoneoscopic and laparoscopic living donor nephrectomy in obese donors, and the impact of donor body mass index on outcome.

**Methods:**

Out of 1108 living donors who underwent hand-assisted retroperitoneoscopic or laparoscopic donor nephrectomy between 2010 and 2018, 205 were identified having body mass index ≥ 30. These donors were included in this retrospective study, analyzing postoperative outcomes and remnant renal function.

**Results:**

Out of 205 donors, 137 (66.8%) underwent hand-assisted retroperitoneoscopic donor nephrectomy and 68 donors (33.2%) underwent laparoscopic donor nephrectomy. Postoperative outcome did not show any significant differences between the hand-assisted retroperitoneoscopic donor nephrectomy group and the laparoscopic donor nephrectomy group in terms of major complications (2.2% vs. 1.5%, *P* = 0.72), postoperative pain scale (4 vs. 4, *P* = 0.67), and the length of stay (3 days vs. 3 days, *P* = 0.075). The results of kidney function in donors after nephrectomy demonstrated no significant differences between the groups. Additional analysis of 29 donors with body mass index ≥ 35 (14.1%) as compared with 176 donors with body mass index 30–35 (85.9%) revealed no significant differences between groups in postoperative outcomes as well as kidney function after donation.

**Conclusion:**

Our results show that laparoscopic living donor nephrectomy for obese donors is safe and feasible with good postoperative outcomes. There were no significant differences regarding postoperative outcome between hand-assisted retroperitoneoscopic and laparoscopic donor nephrectomy. Furthermore, the outcome in donors with body mass index ≥ 35 was comparable to donors with body mass index 30–35.

Kidney transplantation is the most effective option in treating end-stage renal disease. Due to a persistent shortage of deceased donor organs, living donor kidney transplantation is considered as an excellent alternative to expand the kidney donor pool. Careful selection criteria for living donors are warranted because donor nephrectomy can cause complications, including short-term and long-term, in healthy individuals. The body mass index (BMI) is one of the simple parameters used for donor selection. In most transplant centers, a BMI ≥ 35 is considered as a relative contraindication to be a donor [1–4]; however clear cut-off values of BMI are still under debate and further investigations are needed to clarify the significance of donor BMI on outcome not only in donors but also in recipients.

A previous meta-analysis reported that donors with BMI ≥ 30 had significantly longer operative time, higher conversion risk, and increased serum creatinine levels after laparoscopic donor nephrectomy, even though other short-term outcome such as postoperative complications, length of stay (LOS) did not show significantly differences [5]. In contrast, the Organ Procurement and Transplantation Network registry data have shown good outcomes for obese donors (BMI ≥ 30) and their recipients [6]. However, the question whether laparoscopic living donor nephrectomy is safe and feasible in obese donors, and whether there are any differences regarding short-term outcome between hand-assisted retroperitoneoscopic (HARP) and pure laparoscopic (LDN) living donor nephrectomy in obese donors, is unknown.

The aim of this study was to compare the outcome between HARP and LDN in donors with BMI ≥ 30. Furthermore, we evaluated the impact of donor BMI on outcome after living donor nephrectomy.

## Materials and methods

We retrospectively reviewed the kidney transplant database including 1108 consecutive living donors who underwent a donor nephrectomy at the Erasmus MC, University Medical Centre Rotterdam, The Netherlands, between January 2010 and December 2018. Among 1108 living donors, 205 donors with BMI ≥ 30 were extracted. This study was approved by the Ethics Committee of the Erasmus MC, and was conducted in accordance with the tenets of the Declaration of Helsinki.

### Clinical data

For all enrolled donors, the following demographic and clinical data were collected: gender, age, height, weight, BMI, American Society of Anesthesiologists (ASA) physical status, history of hypertension, and relationship between donors and recipients. BMI was categorized into two groups: BMI 30–35 and BMI ≥ 35. Regarding the operative outcome, the type of donor nephrectomy (HARP vs. LDN), the side of the donor nephrectomy (right vs. left kidney), conversion rate to open donor nephrectomy, operative time and blood loss were evaluated. Data on major postoperative complications, mortality, maximum pain scale after surgery until discharge, and the postoperative LOS were recorded as postoperative outcomes. The postoperative major complications were defined as Clavien-Dindo classification grade 3 or higher [7]. Pain scale was evaluated by the Numeric Pain Rating Scale in which a patient selected a whole number (0–10 integers): 0, no pain; 1–3, mild pain; 4–6, moderate pain; 7–10, severe pain [8]. Serum creatinine level (mg/dL) and the estimated glomerular filtration rate (eGFR, mL/min/1.73 m^2^) before donor nephrectomy and after 1 month, 3 month, and 1 year were analyzed.

### Surgical technique

The details of surgical techniques employed were previously described [9, 10]. The selection for LDN or HARP was based on the surgeon preference. Both procedures were performed with the donor placed in right- or left-decubitus position. In LDN, the first trocar was introduced under direct vision, the abdomen was insufflated with carbon dioxide to 14 cmH_2_O pressure and a 30° video endoscope and 3 or 4 additional trocars were introduced. The colon was mobilized and displaced medially, and opening of the renal capsule and division of the perirenal fat was facilitated using an ultrasonic device (Harmonic, Ethicon, Cincinnati, USA). After identification and dissection of the ureter, the renal artery, and the renal vein, a Pfannenstiel incision was made. An endobag (Endocatch, US surgical, Norwalk, USA) was introduced into the abdomen. The ureter was clipped distally and divided. The renal artery and vein were divided using an endoscopic stapler (EndoGia, US Surgical, Norwalk, USA). The kidney was placed in the endobag and extracted through the Pfannenstiel incision.

In HARP, we started with a 7–10 cm Pfannenstiel incision. After blunt dissection to create a retroperitoneal space, a Gelport (Applied Medical, Rancho Santa Margarita, California, USA) was inserted. Blunt introduction of the first trocar between the iliac crest and the handport was guided by the operating surgeon’s hand inside the abdomen through the Gelport. Carbon dioxide was insufflated retroperitoneally to 14 cm H_2_O pressure. Two other 10–12 mm trocars, just outside the midline inferior to the costal margin and in the flank respectively, were inserted to create a triangular shape. For dissection, the aforementioned Harmonic device was used. Dissection of the kidney and dissection and cutting of the renal vessels and ureter were similar to transperitoneal donor nephrectomy but with hand assistance and from a slightly different angle. The kidney was extracted manually and flushed on the back table.

### Statistical analysis

First, donor characteristics and outcome were compared between HARP and LDN groups. Second, the outcome between donors with BMI 30–35 and with BMI ≥ 35 was analyzed. Finally, the outcome of donors with BMI ≥ 30 was compared to those with BMI < 30 using a propensity score matching (PSM). The PSM was conducted using a logistic regression model including preoperative variables with *P* value < 0.20 (6 variables in total), as previously reported [11]. Propensity scores were matched with one-to-one ratio using a caliper width 0.20 of the standard deviation. Data were presented as medians and the interquartile range (IQR) for continuous variables. Categorical data were presented as proportions. Differences between groups were assessed using the Mann–Whitney U-test for continuous variables, and Fisher’s exact test or Chi square test for categorical variables. JMP version 11 software (SAS Institute, Cary, NC) was used for all statistical analyses. A *P* value < 0.05 was considered statistically significant.

## Results

### Study cohort

**Fig. 1:**
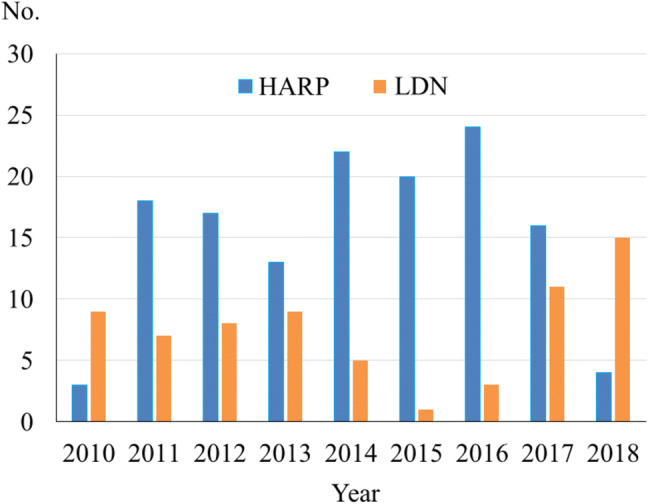
Annual volume of HARP and LDN in obese donors between 2010 and 2018 at Erasmus MC. HARP, hand-assisted retroperitoneoscopic living donor nephrectomy; LDN, laparoscopic living donor nephrectomy

The annual volumes of living donor nephrectomy in donors with BMI ≥ 30 between 2010 and 2018 at the Erasmus MC are shown in Fig. 1. The demographic characteristics of the 205 donors are shown in Table 1. Hypertension was found in 43 donors (21.0%). Regarding the relationship between donors and recipients, 101 (49.2%) were related, 67 (32.7%) were unrelated, 26 (12.7%) were cross over, and 11 (5.4%) were non-directed. Regarding operative outcome, 153 (74.6%) were left and 52 (25.4%) were right donor nephrectomy. Conversion rate to open donor nephrectomy was 0%. The median operative time was 161 min (IQR, 128–193 min) and the median blood loss was 168 mL (IQR, 50–330 mL). The incidence of postoperative major complications was 2.0%. There was no reoperation or mortality after surgery. The maximum pain scale after surgery was 4 (IQR, 2–5). The median LOS was 3 days (IQR, 3–4 days).

**Table 1:** Characteristics between HARP and LDN in donors with BMI ≥ 30

	Total	HARP	LDN	*P* value
No of donors	205	137	68	
Gender (M/F)	79/126	58/79	21/47	0.11
Age (years)	50.5 (41–60.3)	51.8 (42.5–60.7)	48.3 (37.5–58.6)	0.12
Height (cm)	169 (163.5–177)	170 (165–178)	167.5 (162.3–174.8)	0.03
Weight (kg)	93 (85–102.5)	95 (87–104.3)	90 (83–97.8)	0.002
BMI (kg/m^2^)	32.4 (31.1–33.9)	32.6 (31.2–34.1)	31.8 (30.8–33.4)	0.034
ASA (1/2/3)	96/108/1	63/73/1	33/35/0	0.64
Hypertension	43 (21.0)	31	12	0.41
Relationship				
Related	101 (49.2)	66	35	0.047
Unrelated	67 (32.7)	50	17	
Cross over	26 (12.7)	12	14	
Non-directed	11 (5.4)	9	2	
Operative				
Right/left kidney	52/153	29/108	23/45	0.053
Conversion to open	0 (0)	0 (0)	0 (0)	–
Time (min)	161 (128–193)	165 (133–198)	155 (116–190)	0.10
Blood loss (mL)	168 (50–330)	200 (100–400)	67.5 (20–200)	< 0.001
Postoperative				
Major complications (CDc ≥ 3)	4 (2.0)	3 (2.2)	1 (1.5)	0.72
Reoperation	0 (0)	0 (0)	0 (0)	–
Mortality	0 (0)	0 (0)	0 (0)	–
Pain scale (max)	4 (2–5)	4 (2–5)	4 (2.3–5)	0.67
LOS (days)	3 (3–4)	3 (3–4)	3 (2–3)	0.075

Data were presented as numbers (percentages) or median (interquartile range)

*HARP* hand-assisted retroperitoneoscopic living donor nephrectomy, *LDN* laparoscopic living donor nephrectomy, *BMI* body mass index, *ASA* American Society of Anesthesiologists physical status, *CDc* Clavien–Dindo classification, *LOS* length of stay

### Comparison between hand-assisted retroperitoneoscopic versus pure laparoscopic donor nephrectomy in donors with BMI ≥ 30

Perioperative outcomes are also summarized in Table 1. Out of 205 donors, 137 (66.8%) underwent HARP and 68 (33.2%) underwent LDN. BMI was significantly higher in the HARP group than that in the LDN group (32.6 kg/m^2^ vs. 31.8 kg/m^2^, *P* = 0.034). Left donor nephrectomy was more common in the HARP group compared to the LDN group (78.8% vs. 66.2%, *P* = 0.053). The operative time was not significantly different (165 min vs. 155 min, *P* = 0.10); however, blood loss was significantly higher in the HARP group (200 mL vs. 67.5 mL, *P* < 0.001). Postoperative outcome did not show any significant differences between the HARP group and the LDN group in terms of major complications (2.2% vs. 1.5%, *P* = 0.72), pain scale (4 vs. 4, *P* = 0.67), and the LOS (3 days vs. 3 days, *P* = 0.075).

The results of kidney function after nephrectomy are shown in Table 2. Serum creatinine level and the eGFR before nephrectomy and after 1 month, 3 month, and 1 year demonstrated no significant differences between the groups.

**Table 2:** Perioperative kidney function between HARP and LDN in donors with BMI ≥ 30

	Total (*n* = 205)	HARP (*n* = 137)	LDN (*n* = 68)	*P* value
Creatinine (mg/dL)				
Pre	73 (65.5–83)	73 (65.5–82.5)	73 (64.5–83)	0.84
1 month	111 (96–128)	112 (96–129)	108 (96.3–124)	0.61
3 month	109 (95–123)	111 (96–123.5)	105 (92–123.8)	0.56
1 year	106 (93–123)	106 (94–123)	105 (93–123)	0.62
Delta (1 year—pre)	34 (27–42.8)	34 (27–45)	33 (25.5–41)	0.24
eGFR (mL/min/1.73 m^2^)				
Pre	85 (73.5–90)	84 (73–90)	86 (74–90)	0.24
1 month	52 (45–59)	51 (44–58)	53 (46–60)	0.39
3 month	53 (46–61)	52 (46–60)	55.5 (46–63)	0.35
1 year	54 (48–61)	54 (48–61)	55 (48.5–60)	0.79
Delta (1 year—pre)	− 27 (− 33 to − 23)	− 27 (− 33 to − 23)	− 27 (− 33 to − 22)	0.90

Data were presented as median (interquartile range)

*HARP* hand-assisted retroperitoneoscopic living donor nephrectomy, LDN laparoscopic living donor nephrectomy, eGFR estimated glomerular filtration rate

### Comparison between donors with BMI 30–35 versus with BMI ≥ 35

The results of comparison between donors with BMI 30–35 and with BMI ≥ 35 are shown in Table 3. 176 donors (85.9%) were identified having BMI 30–35 and 29 (14.2%) having BMI ≥ 35. No significant differences between the groups were found in terms of demographic characteristics and operative factors. Regarding postoperative outcome, there were no significant differences between the two groups in the incidence of major complications (1.7% vs. 3.5%, *P* = 0.56) and the LOS (3 days vs. 3 days, *P* = 0.68). The results of kidney function showed no significant differences between the groups. At 1 year after nephrectomy, rise in creatinine and decline in eGFR were similar between donor BMI categories.

**Table 3:** Characteristics between donors with BMI 30–35 versus with BMI ≥ 35

	BMI 30–35	BMI ≥ 35	*P* value
No of donors	176	29	
Gender (M/F)	71/105	8/21	0.18
Age (years)	50.5 (41.1–61.3)	49.9 (42.3–55.6)	0.66
ASA (1/2/3)	85/91/0	11/17/1	0.09
Hypertension	34	9	0.17
Operative			
HARP/LDN	116/60	21/8	0.48
Right/left kidney	46/130	6/23	0.52
Conversion to open	0 (0)	0 (0)	–
Time (min)	161 (127–192)	162 (134–209)	0.62
Blood loss (mL)	165 (50–300)	200 (18.5–392)	0.95
Postoperative			
Major complications (CDc ≥ 3)	3 (1.7)	1 (3.5)	0.56
LOS (days)	3 (3–4)	3 (2.5–4)	0.68
Kidney function			
Creatinine (mg/dL)			
Pre	73.5 (66–82.8)	68 (61–83.5)	0.20
1 month	111 (97–128)	104 (88.5–123.3)	0.16
3 month	110 (96–124)	102 (92–122)	0.16
1 year	107 (95–124)	101 (91.5–122)	0.41
Delta (1 year—pre)	34 (27–43)	35 (24–43)	0.60
eGFR (mL/min/1.73 m^2^)			
Pre	85 (73–90)	86 (76–90)	0.82
1 month	51 (44.3–58.8)	52.5 (48.3–63.3)	0.32
3 month	53 (46–60)	56 (47–61)	0.51
1 year	54 (48–61)	54 (47.5–62)	0.72
Delta (1 year—pre)	− 27 (− 33 to − 23)	− 27 (− 31 to − 20.5)	0.58

Data were presented as numbers (percentages) or median (interquartile range)

*BMI* body mass index, *ASA* American Society of Anesthesiologists physical status, *HARP* hand-assisted retroperitoneoscopic living donor nephrectomy, *LDN* laparoscopic living donor nephrectomy, *CDc* Clavien–Dindo classification, *LOS* length of stay, *eGFR* estimated glomerular filtration rate

### Comparison between donors with BMI < 30 versus with BMI ≥ 30 using the propensity score matching

Donor characteristics before and after PSM are demonstrated in Table 4. Donors with BMI ≥ 30 were younger and had higher ASA and more hypertension than donors with BMI < 30. In addition, LDN was much common in donors with BMI < 30 than those with BMI ≥ 30 (77.9% vs 33.2%, *P* < 0.001). After PSM, both groups were well adjusted for matched variables. The receiver- operating characteristic curve area under curve was 0.778. Postoperative outcomes and kidney function were not significantly different between the groups.

**Table 4:** Characteristics of donors with BMI < 30 and BMI ≥ 30: overall and propensity score matching cohort

Variables	Before PSM (*n* = 1108)			After PSM (*n* = 402)		
	BMI < 30	BMI ≥ 30	*P* value	BMI < 30	BMI ≥ 30	*P* value
No of donors	903	205		201	201	
Gender (M/F)	392/511	79/126	0.20	76/125	77/124	0.92
Age (years)	55.0 (42.3–62.8)	50.5 (41.3–60.3)	0.03	52.8 (41.7–60.9)	50.7 (41.6–60.4)	0.58
ASA (1/2/3)	552/347/4	96/108/1	<0.001	91/109/1	96/104/1	0.88
Hypertension	115 (12.7)	43 (21.0)	0.004	37 (18.4)	40 (19.9)	0.70
Operative						
HARP/LDN	200/703	137/68	<0.001	134/67	133/68	0.92
Right/left kidney	288/615	51/154	0.046	53/148	50/151	0.73
Time (min)	152 (126–190)	161 (128–193)	0.17	152 (128–187)	161 (128–194)	0.22
Blood loss (mL)	50 (10–150)	168 (50–330)	<0.001	100 (50–200)	168 (50–330)	0.006
Postoperative						
Major complications (CDc ≥ 3)	7 (0.8)	3 (1.5)	0.38	1 (0.5)	3 (1.5)	0.30
LOS (days)	3 (2–4)	3 (3–4)	0.81	3 (3–4)	3 (3–4)	0.43
Kidney function						
Creatinine (mg/dL)						
Pre	75 (65–84)	73 (65.5–83)	0.21	74 (65.3–82)	73 (66–83)	0.98
1 month	111 (97–127)	111 (96–128)	0.60	111 (98–126)	111 (97–128)	0.80
3 month	110 (97–125)	109 (95–123)	0.51	111 (98–120)	109 (96–124)	0.89
1 year	108 (95–124)	106 (93–123)	0.94	108 (95–120)	107 (93–123)	0.63
Delta (1 year—pre)	33 (27–43)	34 (27–42.8)	0.39	34 (27–43)	34 (27–42.8)	0.66
eGFR (mL/min/1.73 m^2^)						
Pre	84 (73–90)	85 (73.5–90)	0.45	83 (72–90)	85 (73.5–90)	0.48
1 month	52 (45–59)	52 (45–59)	0.87	51 (45–58)	52 (45–58)	0.45
3 month	53 (46–61)	53 (46–61)	0.72	51 (46–59)	53 (46–61)	0.37
1 year	54 (46–62)	54 (48–61)	0.75	53 (46–62)	54 (48–61)	0.76
Delta (1 year—pre)	− 26 (− 32 to − 20)	− 27 (− 33 to − 23)	0.21	− 25 (− 33 to − 19)	− 27 (− 33 to − 23)	0.17

Data were presented as means (standard deviation) or numbers (percentages)

*BMI* body mass index, *PSM* propensity score matching, *ASA* American Society of Anesthesiologists, *HARP* hand-assisted retroperitoneoscopic living donor nephrectomy, *LDN* laparoscopic living donor nephrectomy, *CDc* Clavien–Dindo classification, *LOS* length of stay, *eGFR* estimated glomerular filtration rate

## Discussion

The present study shows the clinical outcome after laparoscopic living donor nephrectomy in a single-center series of 205 donors with BMI ≥ 30 at the Erasmus MC. The results suggest that laparoscopic living donor nephrectomy for donors with BMI ≥ 30 is safe and feasible with good postoperative outcomes including no conversion rate, low major complication risk, and short LOS. In addition, we found no significant differences between HARP and LDN in terms of operative outcome, postoperative outcome and postoperative kidney function except for blood loss. Furthermore, no impact of donor BMI on perioperative outcome and kidney function was found between donors with BMI 30–35 and with BMI ≥ 35 as well as between donors with BMI < 30 and with BMI ≥ 30.

Regarding postoperative outcomes in donors with BMI ≥ 30, all procedures were done laparoscopically with no conversion to open donor nephrectomy and no reoperation after surgery. The outcome was better when compared with previous studies that reported the conversion rate to open procedure with 1.6–2.7% and reoperation with 0.2–0.4% in donors with BMI ≥ 30 [5, 6, 12]. Our institute is one of the largest centers in living kidney donation and transplantation in Western Europe, therefore the beneficial effect of hospital volumes might result in better outcome. Actually several studies have reported that outcomes at higher volume centers are better following laparoscopic donor nephrectomy and kidney transplantation [13–15].

No previous studies have investigated the outcome between HARP and LDN in donors with BMI ≥ 30 so far. Our results demonstrated that operative and postoperative outcomes as well as postoperative kidney function did not show any differences except for intraoperative blood loss. Therefore we believe LDN would be possible even in donors with BMI ≥ 30. Actually 57% of procedures were done by LDN in 2017 and 2018. In contrast, HARP was often conducted at the left kidney (78.8%), although not statistically significant. HARP is considered more challenging at the right side, due to interference of the liver. Furthermore, the use of HARP or LDN did not have any effect on postoperative pain.

With respect to comparison between donors with BMI 30–35 and with BMI ≥ 35, donors with BMI ≥ 35 had comparable operative and postoperative outcomes to donors with BMI 30–35. Furthermore, donors with BMI ≥ 30 had similar postoperative outcomes compared to donors with BMI < 30. These results suggest that laparoscopic living donor nephrectomy for donors with BMI ≥ 30 is safe and feasible when looking at short-term outcome, without compromising outcomes. In addition, kidney function 1 year after donation did not differ significantly across the groups. This result supports a precious report that showed donor BMI was not associated with decline in eGFR and percent change in creatinine level at 6 month after kidney donation [6].

Regarding eligibility criteria for living kidney donor, the CARI guidelines considered obesity (BMI > 30) as a relative contraindication to donation [4]. The British guidelines recommended that individuals with BMI 30–35 should undergo careful preoperative evaluation, and individuals with BMI > 35 should be discouraged from donating because of limited data [16]. The consensus statement of the Amsterdam Forum on the Care of the Live Kidney Donor also recommended that individuals with BMI > 35 should be discouraged from donating, especially when other comorbidities are present [17]. In contrast, The European Association of Urology and the Canadian guidelines have not provided any recommendations for donors with BMI > 30 [18–20]. Accordingly, the question concerning applicable cut-off values of BMI for obese donors remains under debate. A recent systematic review has concluded that the selection of potential kidney donors should not be based on BMI alone, and high BMI should not be considered as an absolute contraindication for living kidney donation. The transplant community should carefully screen each obese individual and make a selection for donation by an obese potential donor with a careful individualized process [21].

Another important issue would be the ethical aspect for long-term outcome after living donor nephrectomy in obese donors. Obese donors (BMI ≥ 30) have been reported to have an increased risk of diabetes mellitus, hypertension, and end-stage renal disease compared to non-obese donors (BMI < 30) during long-term follow-up after donation, although the absolute risk for these outcomes is relatively low [22, 23]. Furthermore, a recent study has reported that obese living kidney donors (BMI ≥ 30) had a 30% increased risk of long-term mortality compared with their non-obese donors (BMI < 30) [24]. Accordingly, we need to select potential obese donors, and pay attention to not only short-term outcome but also long-term outcome of living donor candidates.

Several limitations should be acknowledged in the present study. The present study is a retrospective, single-center analysis, and there may be a potential selection bias for living kidney donors. There were several rationales regarding the selection criteria for LDN or HARP although the final decision was made by the surgeon preference as described above. First female donors were prone to receive LDN because intraabdominal fat is generally less in female donors. Actually 69% of the LDN group were female compared to 58% of the HARP group although not statistically significant. Second we have gained more experiences of LDN in obese donors over the years. Third surgical comfort would be better in LDN compared to HARP. Another concern is long-term outcomes of obese donors. We examined the outcome at 1 year after donor nephrectomy; however, further long-term outcome after nephrectomy is not investigated in this study. The risk of lifestyle diseases and mortality is higher in obese donors (BMI ≥ 30) as we described above, therefore careful long-term follow-up for obese donors is needed, and is provided in our center. In addition, this study does not include recipients’ outcome because we focus on only donor outcome based on the procedure (HARP vs. LDN) and donor BMI. However, a previous study has reported no significant differences in recipient acute rejection, allograft survival, and patient mortality across donor BMI categories [6]. Further prospective large studies are necessary to understand the long-term outcome of recipients from obese donors.

## Conclusions

Our findings suggest that laparoscopic living donor nephrectomy for donors with BMI ≥ 30 is safe and feasible with good postoperative outcomes. There were no significant differences regarding postoperative outcome between HARP and LDN. Furthermore, the outcome in donors with BMI ≥ 35 was comparable to donors with BMI 30–35. BMI itself should not be considered as a contraindication in selection criteria for living kidney donors, based on short-term outcome. However, additional long-term follow-up of donors is needed to examine the impact of donor obesity on outcome including chronic kidney disease and kidney function after living donor nephrectomy.
